# Prognostic factors and the role of primary debulking in operable stage IVB ovarian cancer with supraclavicular lymph node metastasis: a retrospective study in Chinese patients

**DOI:** 10.1186/s12885-024-12215-8

**Published:** 2024-05-06

**Authors:** Chenlian Quan, Xiaojun Chen, Hao Wen, Xiaohua Wu, Jin Li

**Affiliations:** 1 Department of Gynecologic Oncology, Fudan University Shanghai Cancer Center, Shanghai, 200032 China; 2 Department of Oncology, Shanghai Medical College, Fudan University, Shanghai, China

**Keywords:** Ovarian cancer, Stage IVB, Supraclavicular lymph node, Surgery

## Abstract

**Background:**

Recent studies showed heterogeneity in stage IVB patients. However, few studies focused on the prognosis of supraclavicular metastatic ovarian cancer. This study aimed to explore the prognostic factors and the role of primary debulking in IVB ovarian cancer patients with supraclavicular lymph node metastasis.

**Methods:**

We retrospectively analyzed patients newly diagnosed as primary epithelial ovarian cancer with supraclavicular lymph node metastasis from January 2015 to July 2020. Supraclavicular lymph node metastasis was defined as either the pathological diagnosis by supraclavicular lymph node biopsy, or the radiological diagnosis by positron emission tomography-computed tomography (PET-CT).

**Results:**

In 51 patients, 37 was diagnosed with metastatic supraclavicular lymph nodes by histology, 46 by PET-CT, and 32 by both methods. Forty-four (86.3%) with simultaneous metastatic paraaortic lymph nodes (PALNs) by imaging before surgery or neoadjuvant chemotherapy were defined as “continuous-metastasis type”, while the other 7 (13.7%) defined as “skip-metastasis type”. Nineteen patients were confirmed with metastatic PALNs by histology. Thirty-four patients were investigated for BRCA mutation, 17 had germline or somatic BRCA1/2 mutations (g/sBRCAm). With a median follow-up of 30.0 months (6.3–63.4 m), 16 patients (31.4%) died. The median PFS and OS of the cohort were 17.3 and 48.9 months. Survival analysis showed that “continuous-metastasis type” had longer OS and PFS than “skip-metastasis type” (OS: 50.0/26.6 months, PFS: 18.5/7.2months, *p*=0.005/0.002). BRCA mutation carriers also had longer OS and PFS than noncarriers (OS: 57.4 /38.5 m, *p*=0.031; PFS: 23.6/15.2m, *p*=0.005). Multivariate analysis revealed only metastatic PALNs was independent prognostic factor for OS (*p*=0.040). Among “continuous-metastasis type” patients, 22 (50.0%) achieved R0 abdominopelvic debulking, who had significantly longer OS (55.3/42.3 months, *p* =0.034) than those with residual abdominopelvic tumors.

**Conclusions:**

In stage IVB ovarian cancer patients with supraclavicular lymph nodes metastasis, those defined as “continuous-metastasis type” with positive PALNs had better prognosis. For them, optimal abdominopelvic debulking had prognostic benefit, although metastatic supraclavicular lymph nodes were not resected. Higher BRCA mutation rate than the general population of ovarian cancer patients was observed in patients with IVB supraclavicular lymph node metastasis, leading to better survival as expected.

## Background

Ovarian cancer is the most common cause of death in gynecological malignancies worldwide [1]. Approximately 26-30% of patients who were newly diagnosed with ovarian cancer had International Federation of Obstetricians and Gynecologists (FIGO) stage IV disease [2, 3]. The prognosis of stage IVB with distant metastasis was once considered very poor [3]. However, recent studies showed that there was heterogeneity in stage IVB patients, with median survival ranging from 25.2-78.1 months [4–14]. Studies revealed patients with inguinal lymph node metastasis had a relatively favorable prognosis, indicating that part of IVB ovarian cancer patients’ prognosis might not be that dim [10, 11]. However, few studies focused on the prognosis of supraclavicular metastatic ovarian cancer [4, 9, 10].

According to the latest version of FIGO guidelines, patients with advanced ovarian cancer were suggested to receive debulking surgeries by experienced gynecologic oncologists [1]. The importance of optimal debulking for the prognosis of ovarian cancer was well known. However, the extent of debulking surgery for IVB ovarian cancer with supraclavicular lymph nodes metastasis was controversial. Most existing studies defined optimal debulking as no residual tumor in entire body, including distant metastases. There were confusing questions regarding primary surgery for stage IVB ovarian cancer patients. Dose primary cytoreduction have a prognostic significance if complete resection of distant metastasis cannot be achieved? Dose optimal debulking of entire body benefit patients more than optimal debulking of abdominopelvic cavity? Unfortunately, there were no studies answering these questions. Here we presented the study focusing on the debulking of supraclavicular metastatic ovarian cancer patients with the largest sample size, trying to explore the role of surgery and the prognostic factors of this particular group of stage IVB ovarian cancer patients.

## Methods

All patients newly diagnosed as primary epithelial ovarian cancer with supraclavicular lymph node metastasis in Fudan University Shanghai Cancer Center (FUSCC) from January 1, 2015 to July 31, 2020 were retrospectively identified. This study was conducted with the permission of the institutional review board in Fudan University Shanghai Cancer Center. Supraclavicular lymph node metastasis was defined as either the pathological diagnosis by supraclavicular lymph node biopsy, or the radiological diagnosis by positron emission tomography-computed tomography (PET-CT).

Patients who underwent primary debulking surgery (PDS) or neoadjuvant chemotherapy (NACT) followed by interval debulking surgery (IDS) were included consecutively. Those who only received palliative treatments (including palliative chemotherapy or palliative surgery) or underwent primary surgery elsewhere were excluded. If PET-CT suggested the possibility of supraclavicular metastasis but no cancer cells were detected by biopsy, the patient would be excluded from the group. If supraclavicular metastasis was detected by biopsy, whether PET-CT indicated this or not, the patient still would be included.

The retrospective surveillance was done by 4 gynecologic oncologists. They reviewed the electronic medical records from the Medical Records Department of FUSCC. Clinical characteristics of patients collected included ages at diagnosis, histology, metastatic sites, date and extent of debulking surgery, residual tumor status, chemotherapies, dates and sites of recurrence, BRCA status and survival. All pathological results reported by other institutions (sections or paraffin blocks) were rechecked by at least two pathologists from FUSCC. The FIGO system established in 2014 was adopted for staging [14]. Optimal debulking in our study was defined as no macroscopic residual tumor in abdominopelvic cavity after surgery. We divided the patterns of lymph node metastasis into 2 types according to whether there were metastatic PALNs by imaging (PET-CT/CT/MR) before PDS or NACT. “Continuous-metastasis type” was defined as supraclavicular lymph nodes metastasis accompanied by metastatic PALNs. “Skip-metastasis type” was defined as isolated supraclavicular lymph node metastasis without metastasis in PALNs, regardless of another nodal metastasis.

Oncologic outcomes included OS since the diagnosis of ovarian cancer to death and PFS since the diagnosis of ovarian cancer to the first recurrence. Follow-up was completed by telephone follow-up and review of outpatient records. The follow-up period ends on June 30, 2021. No patients were lost to follow-up. Overall survival (OS) was analyzed with Kaplan-Meier curves from the date of primary treatment to the date of death or last follow-up. Progression-free survival (PFS) was analyzed with Kaplan-Meier curves from the date of primary treatment to the date of first recurrence or last follow-up. Survival data in different groups were compared with the log-rank test. Cox proportional hazards regression model was used in multivariate analysis to identify independent variables of survival. Hazard ratios of 95% confidence interval (CI) were also calculated. Distribution frequency of categorical variables was compared with the chi square test. The Statistical Product and Service Solutions (SPSS) v.26 statistical software was performed for all the statistical analyses. The alpha level of statistically significant was considered at 0.05.

## Results

A total of 51 patients who had either histologically or radiologically confirmed supraclavicular lymph node metastasis were included in the study. Among them, 14 (27.5%) patients had supraclavicular lymphadenopathy diagnosed by PET-CT and 37 (72.5%) had histologically confirmed metastasis by fine needle biopsy. The number of patients diagnosed with metastatic supraclavicular lymph nodes was 37 by histology, 46 by PET-CT, and 32 by both methods. A total of 32 patients underwent both PET-CT and SPC fine needle biopsy, whose results of both tests were consistent. Among the rest patients, 5 patients underwent fine needle biopsy and obtained histological confirmation after the possibility of supraclavicular metastasis was discovered by physical examination or CT or B-ultrasound. The rest 14 patients did not undergo needle biopsy and were diagnosed by PET-CT. Table 1 showed the patients’ characteristics and main treatments they received.

**Table 1:** Baseline characteristics of 51 supraclavicular metastatic ovarian cancer patients

**Characteristics**	**N**	**Range/Percentage**
Age, median, years	55	(29-75)
Histology		
High grade serous carcinoma	45	88.2%
Clear cell carcinoma	1	2.0%
Poorly differentiated squamous cell carcinoma	1	2.0%
Adenocarcinoma with difficulty to clarify the specific subtype	4	7.8%
Distant metastasis except nodal metastasis		
Lung	2	3.9%
Bone + liver + lung	1	2.0%
Bone + spleen	1	2.0%
Nodal metastasis other than supraclavicular lymph nodes by imaging		
Intrathoracic (mediastinal, hilar, cardiophrenic angle, diaphragmatic)	21	41.2%
Paraaortic	44	86.3%
Pelvic	29	56.9%
Axillary	6	11.8%
Inguinal	5	9.8%
Treatment		
Primary debulking surgery	22	43.2%
Neoadjuvant Chemotherapy + Interval debulking surgery	29	56.9%
Lymph node dissection		
Paraaortic lymph node dissection (PAND) alone	9	17.6%
Pelvic lymph node dissection (PLND) alone	10	19.6%
PAND+PLND	13	25.5%
Cytoreduction^a^		
Colon resection	4	7.8%
Diaphragmectomy	6	11.8%
Splenectomy	3	5.9%
Partial hepatectomy	2	3.9%
Distal pancreatectomy	2	3.9%
Residual disease in abdominopelvic cavity		
None (R0)	26	51.0%
Residual disease≤1cm	13	25.5%
Residual disease>1cm	12	23.5%
Debulking to no residual in abdominopelvic cavity (R0)		
Primary debulking surgery	9	40.9%
Interval debulking surgery	17	58.6%
Continuous metastasis	22	50.0%
Skip metastasis	4	57.1%
Patterns of recurrence		
Platinum-resistant (recurred within 6 months)	14	34.1%
Platinum-sensitive (recurred above 6 months)	27	65.9%
Mutations in germline or somatic BRCA		
BRCA mutations carrier	17	33.3%
BRCA mutations noncarrier or unknown	34	66.7%

^a^In addition to hysterectomy + bilateral salpingoophorectomy + omentectomy + appendectomy

Twenty-nine patients (56.9%) received median 3 cycles (range 2-10) of NACT and subsequent IDS while the remaining 22 (43.1%) patients received PDS. Since bevacizumab was just approved by national medical insurance for ovarian cancer recently, only 13/51 patients received bevacizumab treatment, of which 8 were in first-line treatment, 1 was in second-line treatment, and 4 were in third-line treatment. For the same reason, only 11/51 patients received poly ADP-ribose polymerase inhibitor (PARPi) treatment, of which 8 were in first-line treatment, 1 was in second-line treatment, and 1 was in third-line treatment. All surgeries were laparotomic and all patients received first-line platinum-based chemotherapy with paclitaxel after debulking surgery. Pathological types included 45 (88.2%) cases of high-grade serous carcinoma, one case (2.0%) of clear cell carcinoma, one (2.0%) of poorly differentiated squamous cell carcinoma and four (7.8%) of adenocarcinoma with difficulty to clarify the specific subtype due to NACT. Four patients (7.8%) had metastasis in distant organs, including lung, bone, liver parenchyma, and spleen parenchyma. Except for supraclavicular lymph nodes, most patients were diagnosed with metastasis in other lymph nodes by imaging (PET-CT/CT/MR) before PDS or NACT. Forty-four patients (86.3%) had metastatic paraaortic lymph nodes (PALNs), 21 patients (41.2%) had metastatic intrathoracic lymph nodes (mediastinal, hilar, cardiophrenic angle, diaphragmatic), 29 patients (56.9%) had metastatic pelvic lymph nodes, 6 patients (11.8%) had metastatic axillary lymph nodes, and 5 patients (9.8%) had metastatic inguinal lymph nodes. Twenty-six patients (51.0%) achieved R0 debulking, which stood for no macroscopic residual tumor in the abdominopelvic cavity. Two patients in the cohort received lymph nodes dissection of the neck after cytoreduction of ovarian cancer (one for primary thyroid cancer and the other for recurrent ovarian cancer). For rest patients in the study, none of them received lymph node resection or primary radiotherapy in supraclavicular lymph nodes area. In the first patient mentioned above, synchronous right thyroid cancer was diagnosed histologically before the surgery for ovarian cancer. Considering that ovarian cancer progresses more rapidly than thyroid cancer, she decided to treat ovarian cancer first. Her postoperative pathology revealed high-grade serous carcinoma with paraaortic lymph node metastasis. After completing chemotherapy, she underwent radical resection of right thyroid cancer with left thyroid lobectomy. The postoperative pathology showed papillary microcarcinoma of the right thyroid gland without right cervical lymph node metastasis, and no tumor was found in the left thyroid gland. Since the left supraclavicular lymph node enlargement did not subside thereafter, the patient strongly requested a left cervical lymph node dissection, pathology of which revealed that 5/64 lymph nodes with metastatic adenocarcinoma tending to be of gynecological origin.

In the whole cohort, 24 patients (47.1%) were investigated for BRCA mutation. Seventeen patients (33.3%) harbored germline or somatic BRCA1/2 mutations (g/sBRCAm), 7 patients (13.7%) were noncarriers, and 27 patients (52.9%) didn’t have records about BRCA mutation. Among the 17 patients with BRCA mutations, 3 harbored both gBRCA1 and sBRCA1 mutations, 1 harbored both gBRCA2 and sBRCA2 mutations, 2 harbored gBRCA1 mutations, 2 harbored sBRCA1 mutations, 3 harbored gBRCA2 mutations, and 6 only recorded as "BRCA positive" with details unknown.

**Fig. 1:**
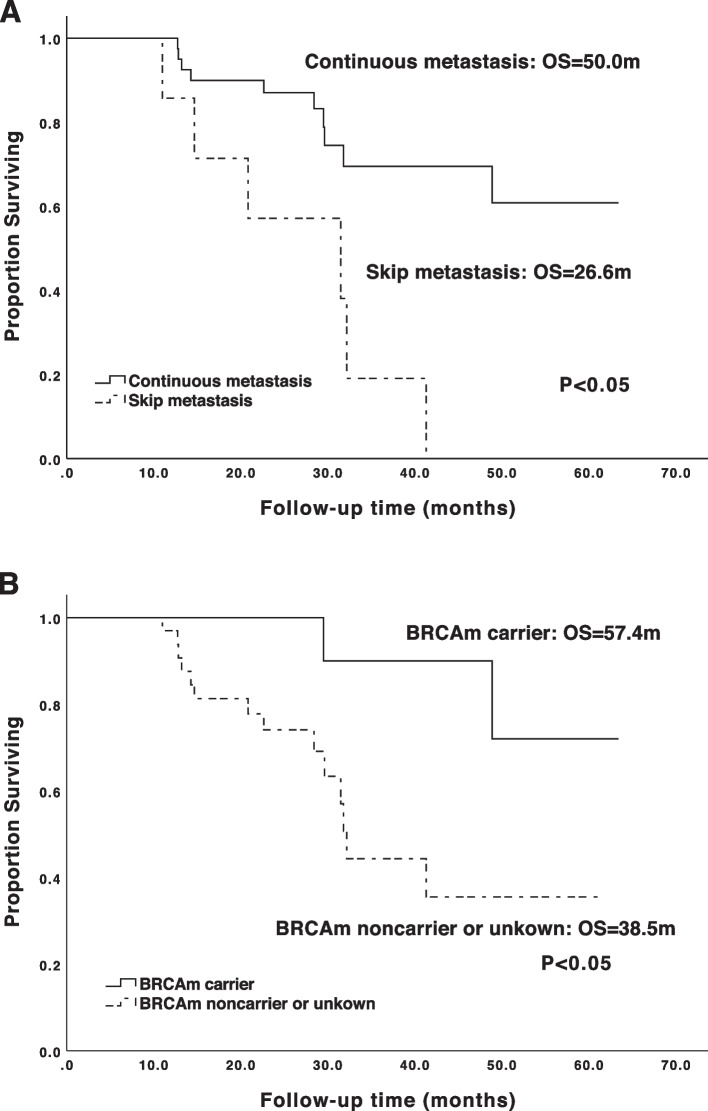
The Kaplan-Meier survival curves for supraclavicular metastatic ovarian cancer patients stratified by different factors. **A** Overall survival in supraclavicular metastatic ovarian cancer patients stratified by types of lymph node metastasis. **B** Overall survival in supraclavicular metastatic ovarian cancer patients stratified by status of BRCA mutations

After a median follow-up time of 30.0 months (range 6.3–63.4), 16 patients (31.4%) died of ovarian cancer. The median PFS and OS were 17.3 and 48.9 months respectively, and the 5-year cumulative survival rate was 47.7% for the whole cohort. A total of 41 patients (80.4%) had disease recurrence. “Continuous-metastasis type” composed 86.3% of the cohort, which was defined as supraclavicular lymph nodes metastasis accompanied by metastatic PALNs in imaging before PDS or NACT. On the contrary, “skip-metastasis type” composed 13.7% of the cohort. The PFS and OS “continuous metastasis type” patients were 18.5 and 50.0 months respectively, both significantly longer than the data of “skip-metastasis type” (7.2/26.6 months for PFS/OS, *p*<0.05) (Fig. 1A). However, in patients with intrathoracic or pelvic lymph node metastasis, no significant differences in OS were found (*p*>0.05). In addition, BRCA mutation carriers had significantly longer OS and PFS than noncarriers (OS: 57.4/38.5 months, *p*<0.05; PFS: 23.6/15.2 months, *p*<0.05) (Fig. 1B).

Multivariate analysis revealed that only metastatic PALNs was independent prognostic factor for OS (*p*<0.05) (Table 2).

**Table 2:** Univariate and multivariate analysis for overall survival in supraclavicular metastatic ovarian cancer patients

**Comparative Factors**	**No. of Patients (%)**	**Univariate Analysis**		**Multivariate Analysis**	
	*N*=51	HR (95% CI)	*p* value	HR (95% CI)	*p* value
Age					
≥60 years	19 (59.4%)	1.283 (0.474,3.475)	0.624	-	-
<60 years	32 (62.7%)				
Distant organ metastasis					
Yes	4 (7.8%)	2.370 (0761,7.376)	0.136	-	-
No	47 (92.2%)				
Paraaortic lymph node metastasis on radiographic imaging					
Yes	44 (86.3%)	0.223 (0.079,0.628)	0.005	0.322 (0.109,0.951)	0.040
No	7 (13.7%)				
Treatment					
NACT + IDS	29 (56.9%)	1.970 (0.681,5.696)	0.211	-	-
Primary debulking surgery	22 (43.1%)				
Residual disease in abdominopelvic cavity after debulking					
Residual disease≤1cm or >1cm	25 (49.0%)	2.473 (0.858,7.126)	0.094	1.941 (0.657,5.730)	0.230
No residual disease (R0)	26 (51.0%)				
Intrathoracic lymph node metastasis on radiographic imaging					
Yes	21 (41.2%)	0.702 (0.243,2.028)	0.513	-	-
No	30 (58.8%)				
Pelvic lymph node metastasis on radiographic imaging					
Yes	29 (56.9%)	1.600 (0.577,4.435)	0.366	-	-
No	22 (43.1%)				
Mutations in germline or somatic BRCA					
BRCAm carrier	17 (33.3%)	5.170 (1.160, 23.036)	0.031	3.118 (0.637,15.247)	0.161
BRCA mutations noncarrier or unknown	34 (66.6%)				

*HR* hazard ratio, *CI* confidence interval, *NACT* neoadjuvant chemotherapy, *IDS* interval debulking surgery, *BRCAm* BRCA mutations

The treatment and prognosis of 7 patients with “skip-metastasis type” were shown in Table 3, while 6 of them have died due to the progression of ovarian cancer.

**Table 3:** Clinical features of 7 “skip-metastasis type” supraclavicular metastatic ovarian cancer patients

	Age, yrs	Histology	Distant organ metastasis	Nodal metastasis except supraclavicular	Treatment	Abdominopelvic residual disease	PFS, months	Follow up, months
1	41	HGSC	/	/	NACT+IDS	R0	6.93	DOD, 10.97
2	29	HGSC	/	Intrathoracic	NACT+IDS	Residual≤1cm	7.20	DOD, 31.47
3	46	HGSC	Lung	Inguinal & pelvic	PDS	Residual≤1cm	15.20	DOD, 32.17
4	54	HGSC	/	Inguinal & axillary	NACT+IDS	R0	5.50	DOD, 14.67
5	61	HGSC	/	Intrathoracic	PDS	R0	20.90	DOD, 41.30
6	61	CCC	Lung, liver & bones	Intrathoracic	NACT+IDS	Residual>1cm	5.80	DOD, 20.83
7	52	HGSC	/	Intrathoracic	PDS	R0	10.30	AWD, 21.53

*PALN* paraaortic lymph node, *PFS* progression-free survival, *HGSC* high grade serous carcinoma, CCC clear cell cancer, *NACT* neoadjuvant chemotherapy, *IDS* interval debulking surgery, *PDS* primary debulking surgery, *AWD* alive with disease, *DOD* died of disease

**Fig. 2:**
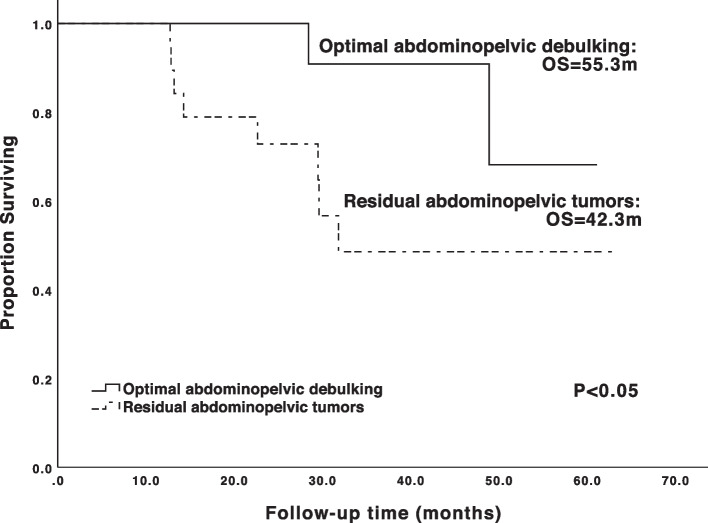
The Kaplan-Meier survival curves for “continuous-metastasis type” supraclavicular metastatic ovarian cancer patients stratified by residual abdominopelvic tumor

Among 44 patients of “continuous-metastasis type”, 22 cases (50.0%) achieved R0 after primary surgery. For the rest 22 patients who did not achieve R0 abdominopelvic debulking, 6 (27.3%) had residual tumor in non-PALN areas, 14 (63.6%) had only residual metastatic PALNs, 2 (9.1%) had residuals in both PALNs and non-PALN areas. For patients with continuous metastasis, those who achieved optimal abdominopelvic debulking had significantly longer OS than those with residual abdominopelvic tumor (55.3/42.3 months, *p*=0.034) (Fig. 2). The same tendency could be observed in PFS but the difference was not statistically significant (22.2/17.3 months, *p*=0.130).

All 17 g/sBRCAm carriers were “continuous-metastasis type”. In the 7 patients of “skip-metastasis type”, no BRCA mutation of any kind was detected (1 was noncarrier and 6 didn’t have records about BRCA mutations). However, no significant correlation between BRCA mutations and continuous-metastasis types was detected (χ^2^=6.220, *p*=0.080).

The two patients with the longest survival in the study had data of 61.1 and 63.4 months. They were both high-grade serous carcinomas with continuous metastasis. One of them had no gross residual tumor in the abdominopelvic cavity after surgery. The other had residual metastatic pelvic lymph nodes of 0.5 cm but had germline BRCA2 mutation. She has been receiving continuous and effective poly ADP-ribose polymerase inhibitor (PARPi) treatment since the last recurrence.

## Discussion

A large number of ovarian cancer patients diagnosed with advanced FIGO stage (III-IV). Generally speaking, patients with stage IVB ovarian cancer have an unfavorable prognosis, with a median OS of 25.2-30.0 months [11]. However, several studies had shown that the survival of ovarian cancer with distant lymph node metastasis as the only evidence for stage IVB was significantly better with a median OS of 39.0-41.1 months [11, 14]. Some authors believed the existing staging system couldn’t fully and accurately predict the biological behavior and prognosis of stage IV ovarian cancer [15]. Patients with parenchymal organ metastasis usually had poor prognosis and should be separated from those who had relatively better prognosis [16]. As for the reason why IVB patients with distant lymph node metastasis had better prognosis, some scholars from MD Anderson Cancer Center [10] believed that this might because of their lower tumor burden in abdominal and pelvic cavity, which lead to higher possibility to achieve optimal abdominopelvic debulking [4]. As evidence, study revealed that for IVB patients with distant lymph node metastasis, the median survival of patients with no macroscopic lesions in omentum was as long as 120 months, while it was only 24 months in patients who had massive lesions in omentum [10]. Some studies also believed that this might because of those patients’ better general condition and tolerance to aggressive debulking and multi-cycle chemotherapy [17]. Our study confirmed the heterogeneity in supraclavicular metastatic ovarian cancer with a large sample size. In addition, when we focused on the group with better prognosis, we found optimal debulking of abdominopelvic cavity in primary surgery played a significant role.

### The prognosis of patients with “continuous-metastasis type” was much better

In our study, patients with supraclavicular lymph nodes metastasis accompanied by metastatic PALNs in imaging (PET-CT/CT/MR) before PDS or NACT were defined as “continuous-metastasis type” while patients without metastasis in PALNs defined as “skip-metastasis type”. We found the median survival of the patients of “continuous-metastasis type” was almost twice as the data of patients with “skip-metastasis type”. Patients with “continuous metastasis-type” accounted for 86.3% of the whole cohort in our study, which was close to the proportion (87.0%) of PALNs metastasis in stage IV ovarian cancer reported in previous literatures [18]. Since some patients did not receive preoperative PET-CT, but only received abdominopelvic CT/MR, chest X-ray and supraclavicular lymph node biopsy, they could not be precisely evaluated for preoperative intrathoracic lymph node metastasis. In that case, we did not adopt intrathoracic lymph nodes as a criterion for judging continuous or skip metastasis. Generally, ovarian cancer that metastasizes to supraclavicular lymph nodes mainly reaches the retroperitoneal para-aortic lymph nodes through the suspensory ligament or pelvic lymph nodes, and then spreads to the upper part of the diaphragm then to the supraclavicular region, through the diaphragmatic and retroperitoneal lymphatic drainage. This "continuous-metastasis" pattern was known as the classic lymphatic drainage pattern of ovarian cancer. However, in some rare cases, cancer cells could metastasize to isolated lymph nodes in distance through blood, showing a pattern of skip metastasis [7]. In additions, peritoneal tumors can drain through diaphragmatic lymphatic vessels to major veins above the diaphragm, which also leads to skip metastasis [1]. Both of the two ways usually imply high burden of tumors and unfavorable biological character of the tumor. Therefore, we speculated that these two anatomical theories might explain why patients defined as “skip-metastasis type” was associated with worse prognosis.

Among the 44 patients with continuous metastasis, suspected PALNs were found in 32 patients during surgery, 22 of whom underwent paraaortic lymph node dissection (PAND) and 16 had no residual metastatic PALNs. Nineteen patients were confirmed with metastatic PALNs by histology. As for the other 10 cases with suspected PALNs, the reason why they didn’t undergo PAND was that their metastatic PALNs were too fixed to be dissected, which means PALNS were closely adhered to main vessels or fixed to the retroperitoneum. Suspected PALNs were not found in 12 continuous metastasis patients during IDS, which might be attributed to the NACT. Therefore, none of them underwent PAND during IDS. Based on the results of this study, we strongly recommend optimal debulking including lymphadenectomy in PDS, while the immunity of lymphadenectomy for those without suspected PALNs in IDS requires further studies.

### Optimal abdominopelvic debulking had prognostic benefit for “continuous metastasis type” patients

The independent factors that reported to affect the prognosis of stage IV ovarian cancer varied, which included: age, physical status, location of metastatic lesions, the volume of ascites, residual tumor after surgery, radical surgery, chemotherapy regimen, etc [3–9, 19–24]. However, the most widely recognized prognostic factor was achieving optimal debulking in primary surgery [4–9, 19–23]. For stage IV patients, to achieve optimal debulking of no residual tumor in entire body sometimes means extremely aggressive and multi-incision surgeries, which may include intrathoracic surgeries [25–28] and lymph node dissections of neck [13, 29]. However, the increased risk of complications and the reduced quality of life should not be ignored in such extensive cytoreductions [26]. At the same time there are different opinions. Some studies proved even with distant metastasis, the main prognostic factor for stage IV ovarian cancer patients was abdominopelvic tumor [24]. Besides, progress in adjuvant therapy could also provide novel treatments for distant metastases postoperatively. In such scenario, optimal debulking of the pelvic and abdominal cavity still benefits patients with distant lymph node metastasis. In our whole cohort, we found that the prognosis of patients who had optimal abdominopelvic debulking was better than those who had suboptimal abdominopelvic debulking, but the difference was not statistically significant (HR = 2.47, 95% CI: 0.86-7.13). We figured the prognostic effect of distant lymph node metastasis might mask the potential benefit of optimal debulking. Therefore, we analyzed the impact of optimal abdominopelvic debulking on prognosis in the subgroup of patients with “continuous-metastasis type”. Our data showed that in patients with “continuous-metastasis type”, the OS of those who achieved optimal abdominopelvic debulking was 55.3 month, significantly longer that the OS of those who were not optimally abdominopelvic debulked (42.3 months, *p*= 0.034). There were studies in which optimal systemic debulking of entire body was done for IVB patients, and the survival data they reported were 25-55 months. [5–9, 19]. Although head-to-head comparisons were not possible, the survival of optimally abdominopelvic debulked patients in our study were not inferior to the survival data reported in literatures. Several theories might explain the prognostic benefit of optimal abdominopelvic debulking. Firstly, most ovarian cancer recurrences were located in abdominopelvic cavity, rather than distant lymph node region [12, 30]. Secondly, compared with the supraclavicular region, recurrences or residual tumors in abdominopelvic cavity were more likely to result in fatal complications, such as intestinal obstruction, cachexia and infection, while supraclavicular tumors seldom cause serious symptoms [3, 24]. In addition, adjuvant therapy might also effectively control the supraclavicular lesions [12]. Optimal systemic debulking of the entire body for stage IV ovarian cancer was never easy, since the rates reported in previous studies never reached 50% [5–9, 19]. Therefore, for stage IV ovarian cancer with supraclavicular metastasis, especially those with PALNs metastasis (“continuous-metastasis type”), we suggested the goal of primary cytoreduction might be no residual tumors in abdominopelvic cavity.

### NACT+IDS vs. PDS

Our study also found that prognosis of patients received PDS was not inferior than those received NACT + IDS (HR = 1.97, 95% CI:0.68-5.70). In fact, the debate on the timing of surgery for stage IV ovarian cancer patients never stopped. In previous retrospective studies comparing the prognosis of IDS and PDS in IVB ovarian cancer patients, results in which PDS were better than, worse than, or equal to IDS were reported separately [4, 12, 31]. Two recent large prospective clinical trials [32, 33] showed that there was no significant difference in survival between stage IV patients received NACT + IDS or PDS, which was similar to our results. Though patients received PDS may had higher rates of complication, our data revealed that if the complications were managed by experienced gynecologic oncologists, patients could achieve equal or even better prognosis than IDS. We believed the main possible advantage of PDS could be, it helped to clarify the extent of tumor, avoid the omission of small lesions after chemotherapy and reduce the resistance to chemotherapy. Larger prospective studies are encouraged to further clarify this issue.

### BRCA mutations

Another factor that had been reported to significantly improve the prognosis of stage IV ovarian cancer was BRCA mutations [30]. Since the genetic testing could not be covered by medical insurance and was only advocated in clinical practice in the recent years in China, 27 patients (52.9%) in our cohort didn’t have records about BRCA mutations, while only 7 patients (13.7%) were recorded as noncarriers. Considering the non-response bias in BRCA status (some “unknown” were actually “negative”), the actual rate of g/sBRCAm in our study was at least 33.3% (17/51), which was already higher than the reported BRCA rate of 28.5% in the largest study of Chinese ovarian cancer patients [34] and 5%-29% mostly in patients from white background [34–40]. Whether it is because patients with BRCA mutations are more prone to supraclavicular lymph node metastasis, or because there is a higher BRCA mutation rate in patients with supraclavicular lymph node metastasis, it is worthy of further studies. It is worth mentioning that we did find 1 patient with gBRCA2 mutation achieved very good OS of 63.4 months. This patient had residual metastatic pelvic lymph nodes of 0.5 cm after primary debulking and received PARPi after her last recurrence which lasted for nearly 2 years. Whether the BRCA mutation rate was higher in IVB stage patients with supraclavicular lymph node metastasis, or the treatment benefit of PARPi can offset the defect of suboptimal debulking of leaving lymph nodes remains to be further studied.

### Limitations

Although we presented the study on supraclavicular metastatic ovarian cancer patients with the largest sample size, there’s no denying our study had some limitations. As a retrospective study, it was difficult to tell whether the survival advantage was due to the successful surgery, or it was the favorable biological nature of the tumor made the optimal debulking possible. In addition, the distribution of BRCA status in this group of patients should be studied in further follow-up. Another limitation was the small sample size, especially the number of patients in the “skip-metastasis type” group due to the rare condition. Since the number of ovarian cancer patients with supraclavicular lymph node metastasis is relatively small, prospective multicenter studies should be encouraged to further verify the findings of this study.

## Conclusion

Our study confirmed that the prognosis of stage IVB ovarian cancer patients with metastatic supraclavicular lymph nodes was heterogeneous. The prognosis of patients of “continuous-metastasis type” who had metastatic PALNs was better than that of “skip-metastasis type” who didn’t have metastatic PALNs. Although metastatic supraclavicular lymph nodes were not surgically removed, patients still benefited from surgeries if optimal debulking were achieved in pelvic and abdominal cavity. Ovarian cancer patients with supraclavicular lymph node metastasis seemed to have a higher BRCA mutation rate than the general population of ovarian cancer patients.

## Data Availability

The data that support the findings of this study are included in the article or available from the corresponding author upon reasonable request.

## Abbreviations

AWD: Alive with disease
CI: Confidence interval
DOD: Died of disease
FIGO: International Federation of Obstetricians and Gynecologists
FUSCC: Fudan University Shanghai Cancer Center
g/sBRCAm: Germline or somatic BRCA mutations
HGSC: High grade serous carcinoma; HR: hazard ratio
IDS: Interval debulking surgery
NACT: Neoadjuvant chemotherapy
OS: Overall survival
PALN: Paraaortic lymph node
PAND: Paraaortic lymph node dissection
PARPi: Poly ADP-ribose polymerase inhibitors
PDS: Primary debulking surgery
PET-CT: Positron emission tomography-computed tomography
PFS: Progression-free survival
PLND: Pelvic lymph node dissection
